# US study of gliding in nondependent lung regions: the dark side of the moon

**DOI:** 10.1186/cc13464

**Published:** 2014-03-17

**Authors:** E De Blasio, M Venditto, A Federico, G Azan, C Pellegrini, C Di Maria, P De Luca, G Rossi

**Affiliations:** 1 Hospital G. Rummo, Benevento, Italy; 2 Hospital San Giovanni Battista, Turin, Italy

## Introduction

A protective ventilatory strategy should prevent VILI, but in patients with larger nonaerated areas hyperinflation may occur during tidal ventilation even during a protective ventilatory strategy [1]. The gliding sign is used as a marker of pneumothorax and, in a study [2], to quantify preoperatively the degree of pleural adhesion in thoracic surgery patients. In our study we measured the variations of gliding (G) and static compliance (Cstat) according to incremental/ decremental variations of PEEP in patients with hypoxic respiratory failure.

## Methods

Ten patients with hypoxic respiratory failure (P/F <300) were ventilated in VcV (Vt of 7 ml/kg, FiO_2 _100%, RR 10/minute); keeping Vt constant, PEEP was gradually increased from ZEEP to 22 cmH_2_O, unless there was occurrence of hypotension or SpO_2 _<90% or Pplat >45 cmH_2_O or G no more visible, and then similarly reduced from 22 cmH_2_O to ZEEP. The gliding was assessed at six points of intercostal spaces bilaterally and the movement of a hyperechoic point of pleura or a b-line was observed during tidal ventilation. For each step, the excursion of G during the inspiratory phase was measured and compared with the Cstat values. Statistical analysis was performed with the Pearson correlation coefficient (PCC).

## Results

**Figure 1:**
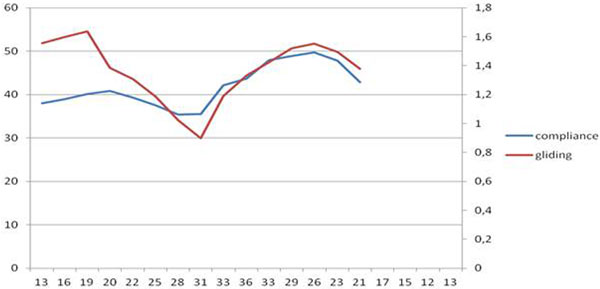
**PCC between mean G (cm) and mean Cstat at different PEEP, in axis Pplat**.

All patients completed the study without adverse events. In all patients we observed a reduction of G and Cstat at the increase of PEEP and specularly an increase of G and Cstat during the reduction of PEEP (Figure 1). In five patients at the lower levels of PEEP (from 0 to 10) an increase of Cstat and G was observed. For all patients the PCC of Cstat and G and was >0.5 (*P *< 0.03), ranging from 0.537 (*P *= 0.017) to 0.964 (*P *< 0.0001).

## Conclusion

The variations of G at different levels of PEEP are consensual with those of Cstat. The study of G during tidal ventilation could help to identify hyperinflation in nondependent lung regions and to optimize lung-protective ventilatory strategies.
